# Impact of β3-adrenergic receptor agonist on tumor progression and metastasis in renal cell carcinoma models

**DOI:** 10.1186/s12935-025-03834-7

**Published:** 2025-06-11

**Authors:** Jee Soo Park, Myung Eun Lee, Minsun Jung, Jongchan Kim, Won Sik Jang, Won Sik Ham

**Affiliations:** 1 Department of Urology and Urological Science Institute, Yonsei University College of Medicine, Seoul, 03722 Republic of Korea; 2 Department of Pathology, Yonsei University College of Medicine, Seoul, Korea; 3 Department of Urology, Yongin Severance Hospital, Yonsei University Health System, Yongin, Korea

**Keywords:** Kidney cancer, Mirabegron, Β3-adrenergic receptor agonist, Tumor immune microenvironment, Perirenal adipose tissue, Fat browning

## Abstract

**Background:**

β3-adrenergic receptor (β3-AR) agonists, widely used in clinical urology, have recently been implicated in modulating cancer progression. While prior studies have reported both pro- and anti-tumor effects via fat browning and immune modulation, the mechanisms and organ-specific outcomes remain unclear. We aimed to confirm the effects of β3-AR agonists on primary tumors and lung metastasis using metastatic orthotopic murine renal cell carcinoma (RCC) models.

**Methods:**

Metastatic orthoptic murine RCC models were developed, and mirabegron, a β3-AR agonist, was orally administered at different dosages and exposure times. The mice were later sacrificed and their kidney and lung tissues harvested. The primary tumor weight and lung nodule number were noted. Perirenal adipose tissue (PAT) browning and tumor immune microenvironment (TIME) remodeling were evaluated and compared between the mirabegron and vehicle treatment groups.

**Results:**

Mirabegron-treated mice showed a significant increase in tumor growth in the early phase; however, tumor growth rates reduced (by > 56%) in the mid and late phases. Mirabegron significantly increased the lung metastatic burden (by > 41%) in all phases. Mirabegron modulated TIME both in primary tumors and lung nodules and increased PAT browning.

**Conclusions:**

The β3-AR agonist increases PAT browning, initially promoting primary tumor progression and possibly contributing to tumor initiation, but eventually inducing immune tolerance, leading to anticancer effects on primary tumors. Effects on lung metastases differed from those on primary tumors.

**Supplementary Information:**

The online version contains supplementary material available at 10.1186/s12935-025-03834-7.

## Background

β3-adrenergic receptor (AR) agonist is commonly prescribed for overactive bladder (OAB) treatment alongside anticholinergics [1]. Unlike anticholinergics, β3-AR agonist has been associated with a significantly lower risk of new-onset dementia in patients [2, 3, 4, 5], a finding supported by recent studies including our own research [6]. Owing to the lower rates of adverse events of β3-AR agonist, it is associated with greater persistence and adherence to compared with anticholinergics [7].

In contrast to these clinical benefits, two recent preclinical studies have reported an association between β3-AR agonist and cancer, although the results regarding whether β3-AR agonist has pro- or anticancer effects are controversial [8, 9]. Sun et al. showed that systemic adipose tissue browning by β3-AR agonists suppresses tumor growth in hepatocellular carcinoma and pancreatic ductal adenocarcinoma models, highlighting the potential for anti-tumor immunity [8]. In contrast, Wei et al. demonstrated that thermogenic activation of perirenal adipocytes enhances clear cell renal cell carcinoma (ccRCC) progression via adipokine secretion and tumor-permissive metabolic remodeling [9]. These conflicting findings, driven by differences in fat depot, cancer type, and immune context, underscore the need for organ- and model-specific investigation. β3-ARs have been recently suggested as potential therapeutic targets in cancers such as melanoma, breast cancer, and colorectal cancer, due to their immunomodulatory roles via regulation of the tumor immune microenvironment (TIME) [10, 11]. The activation of β3-AR, either through exposure to cold or a β3-AR agonist, is involved in the browning process of both brown adipose tissue (BAT) and white adipose tissue (WAT) [12, 13, 14]. Two earlier preclinical studies have focused on the role of β3-AR agonists in adipose tissue browning, reporting conflicting effects on tumor progression [8, 9].

To explore the dual impact of β3-AR agonists on fat metabolism and immune modulation in a relevant disease model, we focused on ccRCC, which exhibits both adipose involvement and immune responsiveness [15, 16, 17]. Perirenal adipose tissue (PAT), a subtype of WAT located between the renal fascia and the renal capsule, has been shown to undergo browning in the context of ccRCC. This browning phenomenon has been associated with enhanced tumor growth and metastasis in recent study [9]. Therefore, it is likely that β3-AR agonists contribute to the progression of ccRCC, and their potential role in tumor initiation warrants further investigation. However, this event has not occurred during clinical development programs for β3-AR agonists [18, 19]. This may be attributed to the modulation of TIME by β3-AR agonists, a mechanism that has not been previously investigated. ccRCC is an immune-responsive tumor frequently associated with loss of tumor suppressors, particularly alterations in the Von Hippel–Lindau gene [20].

However, studies assessing the impact of β3-AR agonists on cancer incidence in perspective of regulation of TIME, especially ccRCC, are lacking. This study aims to delineate the dual roles of β3-AR agonists in regulating adipose tissue metabolism and tumor immunity, using ccRCC as a model that integrates both processes. A deeper understanding of these mechanisms could inform the safe clinical use of β3-AR agonists and potentially guide therapeutic applications in metabolically active, immune-responsive tumors. The kidney and lung were selected to represent contrasting tumor microenvironments: the former being directly influenced by PAT, and the latter providing a fat-independent metastatic site to assess TIME remodeling. Therefore, we aimed to evaluate whether the progression of ccRCC is affected by a β3-AR agonist, and to explore its potential role in tumor initiation using metastatic orthotopic murine RCC models through the perspective of regulation of TIME and PAT browning.

## Methods

### Animals

Seven-week-old male BALB/c (Orient Bio Inc., Seongnam, Korea) and BALB/c nude mice (Central Lab. Animal Inc., Seoul, Korea) were used in this study. The animals were maintained at room temperature and with free access to food and water with 12 h light/dark cycle. Animal experiments were conducted in accordance with the Guide to the Care and Use of Laboratory Animals approved by the Association for Assessment and Accreditation of Laboratory Animal Care and the National Institutes of Health guidelines. The experimental protocol was approved by the Institutional Animal Care and Use Committee (IACUC) of Yonsei University Health System (IACUC No. 2023 − 0197).

### Metastatic orthotopic murine RCC model with mirabegron treatment

We utilized a metastatic orthotopic murine RCC model and orally administered the vehicle or mirabegron. Murine Renca cells were cultured in RPMI 1640 supplemented with 10% fetal bovine serum and 1% penicillin/streptomycin (Gibco, Billings, MT, USA); 1 × 10^5^ cells were directly injected into the kidneys of BALB/c mice. Mice were then divided into two groups: vehicle (control) and mirabegron (treated); *n* = 35 per group (5 predefined time points, 7 mice/time point) for mirabegron 8 mg/kg/day, total *n* = 14 per group (2 predefined time points, 7 mice/time point) for mirabegron 0·8 mg/kg/day and 3·2 mg/kg/day). Mirabegron was administered orally to each mouse at 0·8 mg/kg/day as a conventional dose. For higher doses, we administered 3·2 mg/kg/day and 8 mg/kg/day. Pre-dosing with mirabegron was performed for 28 days before cancer cell implantation. Timepoints for sacrifice—day 9 (early), day 14 (mid), and day 19 (late)—were defined based on the increasing number of lung nodules observed in preliminary experiments. These timepoints were selected to represent distinct phases of metastatic progression rather than primary tumor growth. At predefined time points, the mice were sacrificed, and their kidney and lung tissues were harvested. To visualize lung tumor nodules, their lungs inflated with india ink and white tumor nodules against a black lung background were counted.

### Human RCC xenograft model with co-injected adipocytes

For comparison of the effects between white and brown fat cells on tumor growth, C3H10T1/2 cells were differentiated into matured white or brown adipocytes (1 × 10^6^ cells) and were subcutaneously co-injected together with human clear cell RCC cell line 786-O cells (4 × 10^6^ cells) into the right flank of the BALB/c nude mice (*n* = 6 mice/group). To monitor tumor growth, the tumor diameter was measured every 2 or 3 days with a digital caliper. The tumor volume was calculated using the empirical formula V = 1/2 x [(shortest diameter) ^2^ x (the longest diameter)]. At the end of experiments, tumors dissected from individual mice were weighed, fixed in 4% paraformaldehyde or flash frozen under liquid nitrogen.

### Immunofluorescence staining

Immunofluorescence staining was performed on primary tumors and lung metastatic sites. Frozen tumor tissue sections were blocked with 5% normal goat serum and then incubated overnight with the following primary antibodies: anti-CD31 (Abcam, Cambridge, UK), anti-CD8 (Abcam), anti-PD-L1 (Abcam), anti-Cytokeratin (Santa Cruz Biotechnology, Dallas, TX, USA), anti-Gr-1 (Cell Signaling Technology, Danvers, MA, USA), anti-CD11b (Cell Signaling Technology), anti-F4/80 (Cell Signaling Technology), anti-iNOS (Cell Signaling Technology), anti-CD206 (Cell Signaling Technology), anti-FOXP3 (Cell Signaling Technology), or anti-CD4 (BD Pharmingen, San Diego, CA, USA).

These immune markers were selected to represent key components of the TIME in RCC. CD8 and CD4 were used to assess T cell infiltration, FOXP3 and Gr-1 to characterize immunosuppressive populations such as regulatory T cells (Tregs) and myeloid-derived suppressor cells (MDSCs), and PD-L1 as a clinically relevant immune checkpoint molecule. Macrophage subtypes were evaluated using F4/80, iNOS (M1), and CD206 (M2).

After washing, slides were incubated with fluorescein isothiocyanate-conjugated or Texas Red-conjugated anti-IgG antibodies (Vector Laboratories, Newark, CA, USA). Immunofluorescent images were captured using a Zeiss LSM700 confocal microscope (Carl Zeiss, Oberkochen, Germany). Staining was quantified using the ImageJ software (NIH, Bethesda, MD, USA). The signal intensity was calculated as the number of positively stained pixels relative to the total number of pixels per tumor section (% positive).

### Measurement of uncoupling protein 1 (UCP1) and leptin

In murine samples, total RNA was extracted from primary tumor tissues and PAT obtained from the orthotopic RCC model using TRIzol reagent (Invitrogen, Waltham, MA, USA). RNA was reverse transcribed using the Maxime RT-PCR PreMix Kit (iNtRON Biotechnology, Seongnam, Korea). Quantitative real-time PCR was performed with SYBR Green Master Mix (Applied Biosystems, Waltham, MA, USA) on an ABI StepOnePlus Real-Time PCR System (Applied Biosystems) using specific primers for UCP1 and leptin.

For clinical samples, perirenal adipose tissue was collected from RCC patients who underwent radical nephrectomy. Patients were categorized into two groups: those who had taken mirabegron for ≥ 1 year (*n* = 5) and those who had never taken the drug (*n* = 5). Total RNA was isolated and processed using the same protocol as described above. Data were normalized to 18s rRNA or RPLP0 expression, and relative gene expression was calculated using the 2^ΔΔCT^ method. Primer sequences are listed in Additional File 1. Relative expression levels of UCP1 and leptin were compared between the two groups using Student’s t-test.

### Histopathological evaluation

For hematoxylin and eosin (H&E) staining, primary tumor tissues and PAT were fixed with 4% paraformaldehyde, embedded in paraffin, sectioned (5-µm thick), and then stained with H&E. The slides were evaluated by a pathologist (Minsun Jung) using a light microscope (BX53; Olympus, Tokyo, Japan).

### Western blot

Total proteins were lysed with RIPA lysis buffer, separated on sodium dodecyl sulfate-polyacrylamide gels (SDS-PAGE) and then blotted using the following primary antibodies: anti-UCP1 (Abcam), anti-Leptin (Abcam), or anti-GAPDH (Santa Cruz Biotechnology), and subsequently incubated with horse radish peroxidase (HRP)-conjugated secondary antibody (Cell Signaling Technology). To detect reactive bands, the membranes were examined using the ECL Prime Western Blotting Detection System (Cytiva, Marlborough, MA, USA) and Luminescent Image Analyzer (Amersham ImageQuant 800, Cytiva).

### Statistical analysis

Statistical analyses were performed using GraphPad Prism version 8·0 (GraphPad Software, Inc., La Jolla, CA, USA) and SPSS version 23·0 (IBM Corp., Armonk, NY, USA). All results were expressed as the mean ± standard deviation unless otherwise indicated. Student’s t-test was used unless the dataset did not follow a normal distribution on a Shapiro–Wilk normality test. If the dataset did not follow a normal distribution, the Mann–Whitney U test was used. All statistical tests were two-tailed, and *p*-values < 0·05 were considered significant.

## Results

### β3-AR agonist effects on primary tumors and lung nodules of metastatic orthotopic murine RCC models

**Fig. 1:**
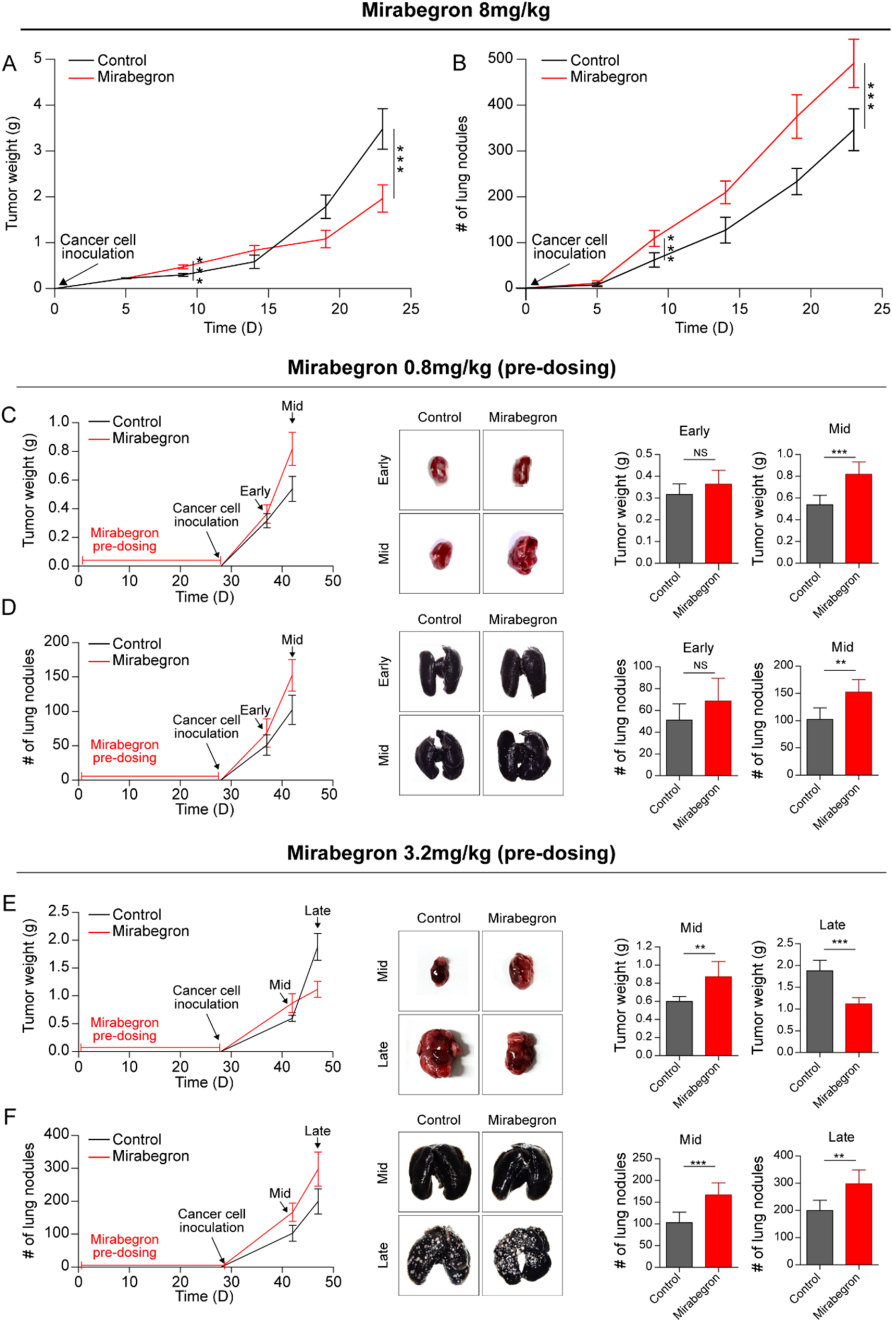
β3-adrenergic receptor agonist effects on tumor growth and metastasis in metastatic orthotopic murine renal cell carcinoma (RCC) models. (**a** and **b**) A dose of 8 mg/kg of mirabegron is administered to tumor-bearing mice. Tumor weights and lung nodules are measured at the indicated time points. (**c** and **d**) A pre-dose of 0.8 mg/kg of mirabegron is administered for 28 days before cancer cell inoculation. Tumor weights and lung nodules are measured on day 9 (Early) and day 14 (Mid) after cancer cell inoculation. (**e** and **f**) A pre-dose of 3.2 mg/kg of mirabegron is administered for 28 days before cancer cell inoculation. Tumor weights and lung nodules are measured on day 14 (Mid) and day 19 (Late) after cancer cell inoculation. Data presented as mean ± standard deviation (SD). *n* = 7 mice at each time point. NS, not significant; *P < 0.05; **P < 0.01; ***P < 0.001

Administration of mirabegron at a dose of 8 mg/kg once daily, which was known to induce adipose tissue browning in previous studies [8, 21], significantly increased tumor growth in the early phase; however, tumor growth rates were eventually reduced in the mid- and late-phases, with more than 56% tumor growth rate reduction (Fig. 1a). An investigation of the number of lung nodules found that mirabegron significantly increased the lung metastatic burden in all phases, with more than 41% increase in metastatic burden (Fig. 1b).

We further investigated the impact of different doses of mirabegron, especially the approved dose of mirabegron 0.8 mg/kg with pre-dosing for 28 days before cancer cell inoculation, generating real clinical settings. Mirabegron significantly induced tumor growth compared to the control at both the primary (Fig. 1c) and lung metastatic sites (Fig. 1d) in the earlier phase. However, during the later phase, β3-AR agonist treatment decreased primary tumor growth and increased lung metastasis. Similar findings were noted at a mirabegron dose of 3.2 mg/kg (Fig. 1e and f).

### Brown adipocytes promote tumor growth in human RCC xenograft model

**Fig. 2:**
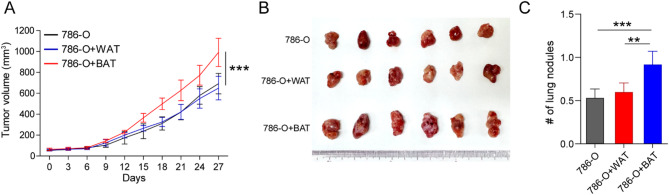
Effect of adipose tissue browning on tumor growth in a human renal cell carcinoma (RCC) xenograft model. (**a**) Tumor growth curves in BALB/c nude mice subcutaneously injected with 786-O cells (4 × 10⁶ cells/mouse) alone or co-injected with either mature white or brown adipocytes (1 × 10⁶ cells/mouse) (*n* = 6 per group). (**b**) Representative images of excised xenograft tumors from each group. (**c**) Final tumor weights measured at the endpoint. Data are presented as mean ± standard deviation (SD). *P < 0.05, **P < 0.01, ***P < 0.001

To validate our findings in human ccRCC context, we performed an in vivo xenograft experiment using 786-O cells co-injected with either mature white or brown adipocytes into BALB/c nude mice. Tumor growth was monitored over time, and the results demonstrated significantly accelerated tumor growth in the group co-injected with brown adipocytes compared to the white adipocyte group (Fig. 2a). At the experimental endpoint, tumors from the brown adipocyte group exhibited greater size and weight (Fig. 2b–c). These findings suggest that brown adipocyte–driven metabolic activation may enhance ccRCC progression, consistent with our observations in the murine model.

### β3-AR agonist-induced PAT browning and high mitotic activity

**Fig. 3:**
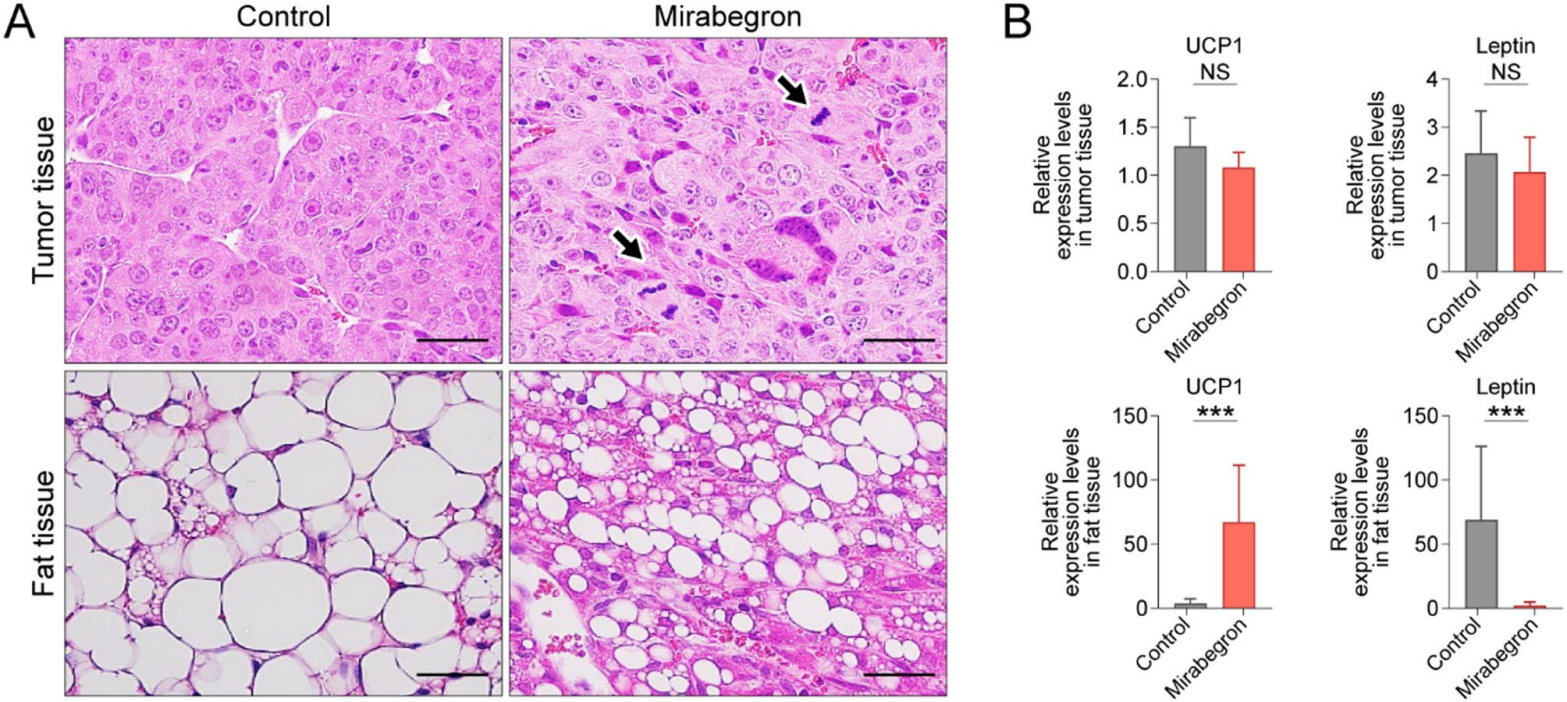
Effects of mirabegron in primary tumor and perirenal fat in metastatic orthotopic murine renal cell carcinoma (RCC) models. (**a**) Hematoxylin and eosin (H&E) histological staining of the tumor and perirenal fat tissue in vehicle- and mirabegron-treated metastatic orthotopic murine RCC model. Black arrows indicate mitotic cells. Scale bar: 50 μm. (**b**) Quantitative real-time reverse-transcription polymerase chain reaction (qRT-PCR) analysis of UCP1 and leptin in tumor and perirenal fat tissues of vehicle- and mirabegron-treated metastatic orthotopic murine RCC model. Data presented as mean ± standard deviation (SD). *P < 0.05; **P < 0.01; ***P < 0.001

The β3-AR agonist induced mitotic activity in primary tumor tissue, compared with the control group (Fig. 3a). Histological analysis of PAT revealed that mirabegron increased the number of smaller multilocular structures compared with the control group (Fig. 3a). Consistently, UCP1 expression in the perirenal fat tissue was increased in the mirabegron-treated group compared to the control group, as demonstrated by both quantitative real-time PCR (Fig. 3b) and western blot analysis (Additional File 2). These results provide transcriptional and protein-level evidence that β3-AR agonist treatment promotes adipose tissue browning in fat tissue.

### Adipose browning in RCC patients treated with mirabegron

To evaluate the clinical relevance of adipose tissue browning, we also analyzed perirenal adipose tissue samples from RCC patients who had undergone radical nephrectomy. Patients were grouped by mirabegron exposure: ≥1 year of use (*n* = 5) versus no use (*n* = 5). Quantitative PCR analysis revealed that UCP1 expression was significantly elevated in the mirabegron group (*P* = 0.001), while leptin expression was significantly decreased in the mirabegron group (*P* = 0.023) (Additional File 3). These findings support that chronic mirabegron use may promote fat browning in humans, in line with our preclinical results.

### β3-AR agonist-induced TIME modulation in primary tumor and lung nodules

**Fig. 4:**
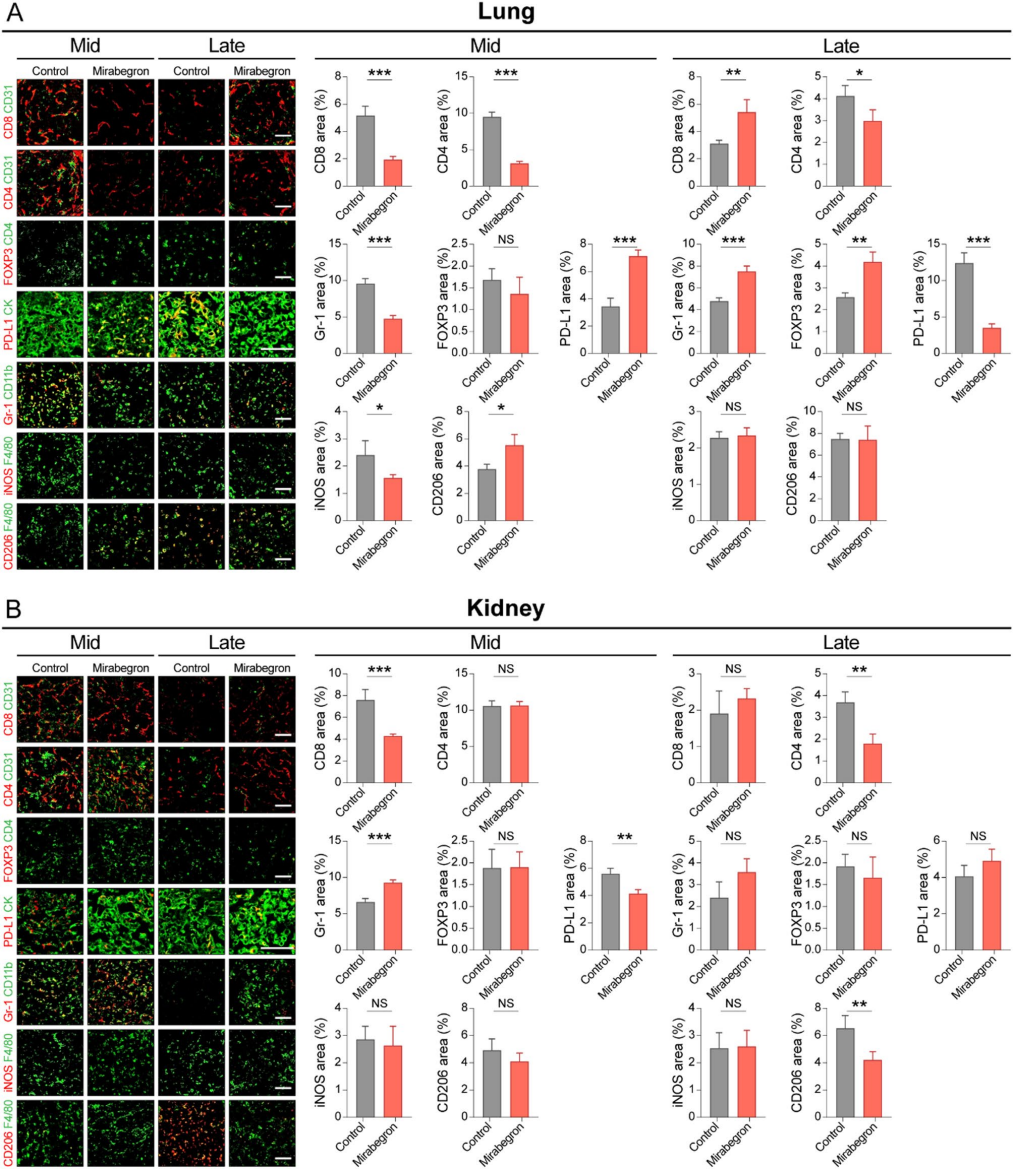
Association between a β3-adrenergic receptor agonist and the tumor immune microenvironment of primary tumor and lung metastasis in metastatic orthotopic murine renal cell carcinoma (RCC) models. (**a**) Immunofluorescence staining of metastatic lung tumor tissues from vehicle- and mirabegron-treated metastatic orthotopic murine RCC model. (**b**) Immunofluorescence staining of kidney tumor tissues from vehicle- and mirabegron-treated metastatic orthotopic murine RCC model. Representative images and quantitative analyses are shown. Scale bar: 50 μm. Quantification of CD8 + cytotoxic T cells, CD4 + helper T cells, Gr-1 + myeloid-derived suppressor cells, FOXP3 + regulatory T cells, PD-L1+, iNOS+, and CD206 + tumor-associated macrophage signals (random fields per group). Data presented as mean ± standard deviation (SD). NS, not significant; *P < 0.05; **P < 0.01; ***P < 0.001

We investigated whether the β3-AR agonist displayed both pro- and anticancer effects on ccRCC by modulating the TIME (Fig. 4). Drastic TIME changes were noted in the lung nodules (Fig. 4a), where the β3-AR agonist increased CD8 + T cell recruitment by 2.9-fold, while there was a 1.7-fold decrease of CD8 + T cells in the control group. In addition, MDSCs and Tregs were increased by 1.6-fold and 3.1-fold, respectively, by the β3-AR agonist. In contrast, a 2.0-fold decrease or no significant increase (1.5-fold increase) was noted in the control group. A 2.1-fold downregulation of PD-L1 was observed in the β3-AR agonist group, while a 3.7-fold upregulation of PD-L1 was observed in the control group.

For the TIME of primary ccRCC cells, the β3-AR agonist significantly induced the polarization of macrophages from the M2 to M1 phenotype compared to the control group (Fig. 4b).

## Discussion

In this study, a β3-AR agonist has shown both pro- and anti-cancer effects depending on the organ site—kidney (primary tumor) versus lung (metastasis)—and the timing of exposure. We observed that β3-AR agonist treatment had opposing effects depending on organ site and timing, with early promotion and later suppression of primary tumor growth, and persistent enhancement of lung metastasis.

These contrasting effects led us to consider multiple, context-dependent mechanisms underlying the observed outcomes. We hypothesized that the effects of the β3-AR agonist are influenced by both modulation of TIME and adipose tissue browning, although additional mechanisms may also contribute. Previous studies have only investigated the role of adipose tissue browning in cancer progression [8, 9]. However, since recent studies reported the role of β3-AR agonists in the regulation of TIME [10, 11], we evaluated the TIME of primary tumors and lung nodules after β3-AR agonist treatment. Since TIME and the location of fat are different by organs, we believe that the effect of β3-AR agonists would be different according to different organs even if the cancer cells originated from the same cancer type. Therefore, we have used metastatic orthotopic murine RCC models to evaluate this difference. β3-AR signaling has emerged as a contributor to cancer development and progression [10]. In melanomas, β3-AR blockade reduced tumor volume and the development of tumor vasculature through decreased cell proliferation and increased apoptosis of melanoma cells [22, 23, 24]. However, this did not apply to all types of cancers. The differences in the effects of β3-AR agonists on each cancer could be mainly due to the different locations of nearby fat depots according to cancer type [25] and characteristics of the TIME of each cancer [26].

Two recent preclinical studies have reported an association between fat browning and cancer [8, 9]. Wei et al. demonstrated that PAT browning enhances ccRCC growth, invasion, and metastasis, showing that the suppression of adipocyte browning enhanced the antitumor efficacy [9]. In contrast, Sun et al. reported that browning of fat by a β3-AR agonist is essential for tumor suppression [8]. These two contradictory results were all demonstrated in our model, where we investigated different organs, such as the kidneys and lungs, and observed different effects of the β3-AR agonist.

The lungs have no distant fat deposits that can directly influence cancers through fat browning. Therefore, lung cancers must be approached using the concept of TIME. In our models, the β3-AR agonist induced a significant increase in MDSCs, Tregs, and CD8 + T cells in the lung nodules. Calvani et al. demonstrated that blocking of β3-AR reduced melanoma growth, and this effect was concomitant with a significant increase in NK and CD8 + T cells and a strong reduction in Tregs and MDSCs within the tumor mass [27]. For melanomas and colorectal and breast cancers, a high density of CD8 + T cells represents a good prognosis [26]. However, in RCC, CD8 + T-cell density is associated with a poor prognosis [26] Therefore, as in our study, it is plausible that an increase in CD8 + T cells results in the progression of lung metastasis. Furthermore, an increase in the number of Tregs and MDSCs results in immune suppression, which further aggravates lung metastasis. In primary ccRCCs, the shift from M2 to M1 macrophage polarization by mirabegron induces an anti-cancer environment [28], eventually reducing primary tumor growth.

However, the results of our study demonstrated that the β3-AR agonist initially induces RCC. This is consistent with the findings of Wei et al., in which PAT browning enhanced ccRCC growth, invasion, and metastasis [9]. To further examine the role of adipose browning in RCC progression, we performed an in vivo xenograft experiment using the human ccRCC cell line 786-O co-injected with either white or brown adipocytes. Although this model did not involve mirabegron treatment, tumors in the brown adipocyte group exhibited significantly greater growth, supporting the notion that adipose browning itself may promote RCC progression, independent of β3-AR agonist administration. In addition, to explore the clinical relevance of adipose browning, we analyzed perirenal fat tissues from RCC patients who had undergone radical nephrectomy. Patients who had been treated with mirabegron for over one year exhibited significantly higher UCP1 expression compared to non-users, while leptin levels were unchanged. These findings support the notion that chronic mirabegron exposure may induce adipose browning in humans, consistent with our preclinical and xenograft observations. As UCP1 is highly upregulated in adipocytes of activated BAT and browning WAT [29], we concluded that the β3-AR agonist significantly induced an increase in UCP1 expression levels in PAT but did not affect tumor tissue in our study. Lung metastasis was not affected by fat browning because of the distant location of fat depots. Kidney cancer is closely associated with PAT, and the effect of β3-AR agonists on browning primarily originates from PAT. However, in other cancers, the major sources of fat browning differ. Although Sun et al. reported that a β3-AR agonist displayed potent anti-cancer effects, their study used only pancreatic ductal adenocarcinoma and hepatocellular carcinoma models [8]. Moreover, there are intrinsic species-specific differences between humans and rodents, such as differences in the proportion of different β-AR subtypes, intrinsic thermogenic activity, and BAT depots [30]. Importantly, in the kidney, mirabegron treatment led to a significant reduction in tumor growth during the mid-to-late stages, suggesting a potential tumor-suppressive effect with prolonged exposure. This contrasts with its consistent effect of promoting lung metastasis, highlighting how the biological impact of β3-AR agonists can vary by organ site and timing. Therefore, it is an overgeneralization that mirabegron has anti-cancer effects. Further studies are required to clarify the discrepancies between cancer types and the effects of β3-AR agonists. Further mechanistic validation, such as gene silencing or pathway inhibition studies, will be necessary to confirm the causal roles of TIME remodeling and adipose tissue browning.

In parallel with the preclinical experiments presented here, we are currently conducting a retrospective cohort study to investigate whether mirabegron use is associated with increased metastasis in patients with RCC. The results of this clinical analysis will provide valuable insight into the translational relevance of our findings.

## Conclusions

This is the first study to compare the effects of a β3-AR agonist according to different organs and exposure time to the β3-AR agonist using metastatic orthotopic murine RCC models. In line with the growing awareness of the importance of fat browning and modulation of TIME, especially by mirabegron, in cancer, our findings show that mirabegron has both risks and benefits in association with RCC progression and may influence tumor initiation, supporting further investigations into the regulation of fat browning and TIME by β3-AR agonists in real clinical settings.

## Electronic supplementary material

Below is the link to the electronic supplementary material.

Supplementary Material 1

## Data Availability

No datasets were generated or analysed during the current study.

## Abbreviations

AR: Adrenergic receptor
OAB: Overactive bladder
TIME: Tumor immune microenvironment
RCC: Renal cell carcinoma
ccRCC: Clear cell RCC
PAT: Perirenal adipose tissue
WAT: White adipose tissue
BAT: Brown adipose tissue
UCP1: Uncoupling protein 1
H&E: Hematoxylin and eosin
MDSCs: Myeloid derived suppressor cells
Tregs: Regulatory T cells
