# Supplementary material for: Impact of β3-adrenergic receptor agonist on tumor progression and metastasis in renal cell carcinoma models
Source: Cancer Cell Int. 2025 Jun 11;25:209. doi: 10.1186/s12935-025-03834-7 (PMC12153155; doi:10.1186/s12935-025-03834-7)
Supplement: Supplementary file 1 — Supplementary Material 1 [file 12935_2025_3834_MOESM1_ESM.docx]

**Impact of β3-Adrenergic Receptor Agonist on Tumor Progression and Metastasis in Renal Cell Carcinoma Models**

Jee Soo Park, Myung Eun Lee, Minsun Jung, Jongchan Kim, Won Sik Jang, Won Sik Ham

**Additional File 1. List of primer sequences used for qRT-PCR**

| **qRT-PCR primers** | |
| --- | --- |
| **Gene (Mouse)** | **Primer sequence** |
| UCP1 | F: CTTTGCCTCACTCAGGATTGG |
|  | R: ACTGCCACACCTCCAGTCATT |
| Leptin | F: CAGGATCAATGACATTTCACACA |
|  | R: GCTGGTGAGGACCTGTTGAT |
| 18s | F: AGTCCCTGCCCTTTGTACACA |
|  | R: CGATCCGAGGGCCTCACTA |
| **Gene (Human)** | **Primer sequence** |
| UCP1 | F: GCAGGGAAAGAAACAGACCCT |
|  | R: ACTTTCACGACCTCTCTCGG |
| Leptin | F: TGCCTTCCAGAAACGTGATCC |
|  | R: CTCTGTGGAGTAGCCTGAAGC |
| RPLP0 | F: TGGTCATCCAGCAGGTGTTCGA |
|  | R: ACAGACACTGGCAACATTGCGG |

Abbreviations: qRT-PCR: quantitative reverse transcription polymerase chain reaction; UCP1: uncoupling protein 1

**Additional File 2. Western blot analysis of UCP1 and leptin protein expressions in fat tissue from mirabegron- and vehicle-treated mice.**


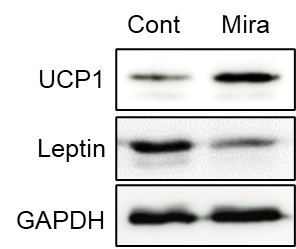


**Additional File 3. UCP1 expression is increased in perirenal adipose tissue of RCC patients treated with mirabegron. Data presented as mean ± standard deviation (SD). **P* < 0.05; ***P* < 0.01; ****P* < 0.001.**
